# Globe salvage treatment in group D and group E retinoblastoma

**DOI:** 10.22336/rjo.2021.5

**Published:** 2021

**Authors:** Hussain Ahmad Khaqan, Rahul Rachwani Anil, Carlos Rocha de Lossada, Francisco Zamorano Martín, María García Lorente, Flavia Pennisi, Chiara Bonzano, Davide Borroni

**Affiliations:** * Ameer Ud Din Medical College, Lahore General Hospital, Post Graduate Medical Institute, Lahore, Pakistan; ** Department of Ophthalmology, Regional University Hospital of Malaga, Malaga, Spain; *** Departamento de Oftalmología, Hospital Costa del Sol, Marbella, Spain; **** Department of Ophthalmology, University Vita-Salute, IRCCS Ospedale San Raffaele, Milan, Italy; ***** Eye Clinic, DiNOGMI, University of Genoa and IRCCS San Martino Polyclinic Hospital, Genoa, Italy; ****** The Veneto Eye Bank Foundation, Venice, Italy

**Keywords:** retinoblastoma, intravenous chemotherapy, intravitreal Melphalan, globe salvage

## Abstract

**Importance:** Globe salvage marks the treatment success of retinoblastoma.

**Background:** To evaluate four treatment strategies in group D and group E retinoblastoma.

**Design:** Retrospective case series in a tertiary hospital.

**Participants:** 81 patients with Group D and Group E retinoblastoma.

**Methods:** Participants were divided into four sets. In set I, eyes received primary intravenous chemotherapy (IVC), cryotherapy (CT), laser therapy (LT) and Intravitreal Chemotherapy with Melphalan (IViC). In set II, primary IVC was combined with second line IVC, CT, LT and IVT-M. Set III eyes received primary IVC and Intra-arterial chemotherapy (IAC), CT, LT and IViC. Set IV eyes received IAC, CT, LT and IViC. Treatment failure was defined as inadequate response during or after IVC or IAC.

**Main Outcome Measures:** globe salvage and enucleation rates.

**Results:** 52 eyes were included in group D and 29 in group E. In group D, globe salvage was obtained in 8 out of 11 eyes in Set I, 13 out of 19 eyes in set II, 5 out 6 eyes in set III, and 13 out of 16 eyes in set IV. In group E, enucleation was performed in 17 eyes. Global salvage was obtained in 0 out of 2 eyes in set I, 2 out of 3 eyes in set II, 3 out of 5 in set III, and in 1 out of 2 eyes in set IV.

**Conclusions:** IVC with adjuvant IAC, LT, CT and IViC has shown favorable results as a treatment method for group D and group E retinoblastoma.

## Introduction

Retinoblastoma treatment has experienced changes over the years and a wide range of options are currently available. Over the past two decades, intravenous chemotherapy (IVC) has been the mainstay of the treatment in addition to adjuvants if required. 

The Reese-Ellsworth (R-E) classification system for intraocular retinoblastoma [**1**], which predicts the prognosis after radiotherapy, has been outdated. Presently, the International Retinoblastoma Classification (ICRB) [**2**], which predicts the treatment response to chemotherapy, is preferred. According to the ICRB, most eyes with Group E disease typically end in enucleation, while groups A to C have demonstrated success in approximately 90% of the cases [**2**].

With the introduction of IVC, external beam radiotherapy (EBRT) was widely abandoned and IVC has been the primary treatment for group D eyes in many centers [**3**], with globe salvage rate up to 47% [**4**]. However, globe salvage is challenging in groups D and E when relying on IVC alone.

Recently, intra-ophthalmic artery chemotherapy (IAC) [**5**,**6**] has been popularized as a primary treatment for group D eyes due to its selectivity and reported success in achieving tumor control. Moreover, it has been used in some centers for bilateral cases [**7**]. However, first-line IAC has possible drawbacks as it is selective to the eye, hence being a potential risk of systemic spread [**8**,**9**]. In addition, it requires advanced infrastructure and expertise. Efforts to preserve the eye without compromising patient survival are increasingly favored. 

The purpose of this study was to report the management course and outcomes of group D and E retinoblastoma patients treated in our center with IVC, and a combination of available adjuvant therapeutic strategies.

## Material & methods

Eighty-one eyes classified as group D and group E retinoblastoma were included in this retrospective, observational and longitudinal study. Patients underwent treatment from January 2015 to December 2019 in the Department of Ophthalmology, Lahore General Hospital, Lahore, Pakistan. All patients included in this work were adequately informed verbally and in writing of the benefits, characteristics, and risks of surgeries. All patients signed a consent form prior to the surgery and after the interview with the ophthalmologist. This study was conducted in accordance with the tenets of the Helsinki Declaration and obtained approval from the Ethics Committee of Lahore General Hospital. 

Inclusion criteria were defined as group D or group E retinoblastoma. All patients received a combination of cryotherapy, laser therapy, intravitreal chemotherapy with Melphalan (IViC) and one of the four treatment regimens as it follows: set I included primary intravenous chemotherapy (IVC), set II comprised patients treated with primary and second line IVC, set III was defined as a combination of primary IVC and intra-arterial chemotherapy (IAC), and set IV received IAC only.

Primary IVC consisted of six cycles of intravenous vincristine, etoposide, and carboplatin (VEC) every 3 weeks. Early treatment failure was defined as inadequate response of the main tumor or seeding, or drug intolerance, requiring change in management during or immediately after IVC cycles. Treatment response was assessed in terms of size, color, vascularization, calcification degree, tumor focality and density. These factors determined if a change in the management plan was required.

In such cases, a second line IVC was carried out, and included four to six cycles of ifosfamide, vincristine, and doxorubicin (IVAd) or IAC. Intra-arterial chemotherapy was administered using Melphalan or Topotecan, as reported in previous studies [**10**]. Intravitreal Melphalan was used according to the present guidelines [**11**]. Acrylic implants, with the formerly described myoconjunctival technique, were carried out in enucleated eyes [**12**].

SPSS version 23 was used for statistical analysis of the data. Chi-square test function was used to compare outcomes across different treatment strategies. Kaplan-Meier survival analysis estimated globe salvage expectancy.

## Results

Mean age of patients was 27.31 ± 11.83 months at presentation. Fifty-two (64.20%) eyes presented with group D disease and 29 (35.80%) eyes had group E disease, classified according to the International Classification of Retinoblastoma (ICRB).

**Group D**

Mean age of patients in group D (n=52) was 30 ± 12.29 months. They were distributed in the four treatment sets, priorly defined, as it follows: Set I included 11 eyes (21.15%), Set II - 19 eyes (26.54%), Set III - 6 eyes (11.54%) and Set IV - 16 eyes (30.77%). 

Treatment outcomes for set I were 8 eyes (72.73%) salvaged, and 3 eyes (27.27%) were enucleated. In set II, 13 eyes (68.42%) showed good response, and 6 eyes (31.58%) were enucleated. In set III, 5 eyes (83.33%) out of 6 were saved, while 1 eye (16.67%) showed treatment failure and resulted in enucleation. In set IV, 3 eyes (18.75%) out of 16 were enucleated and globe salvage was accomplished in 13 eyes (81.25%). Chi-square test demonstrated similar outcomes between different treatment sets [χ2 (4, N=52) =1.03, p=0.795)]. 

**Group E**

Mean age of patients in group E (n=29) was 22.48 ± 9.35 months. Upfront enucleation was performed in patients with unilateral disease and decline of function with treatment intolerance, and occurred in 17 eyes (58.62%). The remaining 12 eyes (41.38%) were distributed as it follows: set I consisted of 2 eyes (16.67%), set II of 3 eyes (25%), set III of 5 eyes (41.67%), and Set IV of 2 eyes (16.67%). In set I, both eyes (100%) were enucleated due to treatment failure. In set II, out of 3 eyes, 2 eyes (66.67%) showed good response and 1 eye (33.33%) was enucleated. In set III, 2 eyes (40%) were enucleated, while 3 eyes (60%) were salvaged. In set IV, globe salvage was observed in 1 eye (50%), whereas 1 eye (50%) ended in enucleation. Fisher’s exact test demonstrated similar outcomes between different treatment sets (p=0.740).

In group D, 39 (75%) out of 52 eyes were salvaged, while only 6 (20.68%) out of 29 eyes in group E were salvaged. Logistic regression analysis confirmed significant differences in success rates between treatment outcomes in group D and group E (p=0.001). Combined success rates of separate treatment sets for both groups did not show statistical differences (χ2(4, N=64) =1.04, p=0. 792). There was a statistically significant difference in the mean age between group D and group E (p=0.002). 

There were no cases of metastatic spread from intraocular retinoblastoma and no deaths were reported. Overall treatment failure occurred in 13 eyes (25%) in group D, and 6 eyes (50%) in group E, all of which were subjected to secondary enucleation. Given the inadequate treatment response, the treatment method was changed during or immediately after IVC. Results are summarized in **Table 1**.

**Tabel 1:** Summary of treatment results for each set and group

Treatment set	Group D		Group E		Total	
	Salvaged	Enucleated	Salvaged	Enucleated	Salvaged	Enucleated
Set I – primary IVC, CT, LT, IViC	8 (72.73%)	3 (27.27%)	0 (0%)	2 (100%)	2 (100%)	5 (38.46%)
Set II – primary and second line IVC, CT, LT, IViC	13 (68.42%)	6 (31.58%)	2 (66.67%)	1 (33.33%)	15 (68.18%)	7 (31.81%)
Set III – IVC+IAC, CT, LT, IViC	5 (83.33%)	1 (16.67%)	3 (60%)	2 (40%)	8 (72.72%)	3 (27.27%)
Set IV – IAC, CT, LT, IViC	13 (81.25%)	3 (18.75%)	1 (50%)	1 (50%)	14 (77.78%)	4 (22.22%)
Total	39 (75%)	13 (25%)	6 (50%)	6 (50%)	45 (70.31%)	19 (29.69%)
IVC = intravenous chemotherapy, CT = cryotherapy, LT = laser therapy, IViC = intravitreal Melphalan						

## Discussion

In last three decades, there has been a tremendous advancement in the management of retinoblastoma, and several new approaches are currently available. These advancements resulted in the change of the R-E classification of intraocular retinoblastoma, that predicted globe salvage in the era of EBRT [**1**], to the ICRB, which establishes treatment success in the chemotherapy era [**2**]. This study bridges the period in our department after the establishment of IVC for retinoblastoma [**4**,**14**-**16**], to the emergence of IAC for globe salvage. Our cohort consisted of 52 group D and 29 group E retinoblastoma eyes followed up for a median time of nearly 5 years, and an eye salvage rate of 75% for group D, and 20.68% for group E retinoblastoma. This is significantly higher than in previous similar studies [**3**,**17**-**25**]. Before the advent of IAC, Shields et al. [**3**] reported 47% globe salvage rate in group D eyes treated by primary IVC, which was the highest success rate achieved in the pre-IAC era. However, Shields et al. [**3**] used the Philadelphia version of the IRCB, whereas we used a slightly different criterion to define group D eyes. We classified the latter according to the Children’s Hospital Los Angeles scheme [**2**], and it was shown that such discrepancies may adversely affect attempts to compare treatment outcomes [**26**]. Nevertheless, this does not fully explain the disparity in salvage rates between the two studies.

A study of 18 eyes with retinoblastoma, conducted between 1995 and 2003, reported a 61% success rate with primary IVC and adjuvant EBRT [**23**]. Since then, the role of EBRT has been reserved for treatment failure, especially due to the increased risk of secondary cancers in patients with germline retinoblastoma [**27**]. In addition to the known risk derived from EBRT, these findings reinforce that EBRT has a declining role in the treatment of retinoblastoma. IAC injections and, more recently, IViC injections, have proven to be important adjuvant therapies as they play an important role in achieving relatively high survival rates in this study. Both were initially used as a rescue treatment in more resistant cases as adjuvants to IVC.

Yousef et al. [**8**] reported a globe salvage rate of 67% after IAC as a second-line treatment for all ICRB groups. However, our study focused on group D and E disease only. In their study [**8**], IAC showed a success rate of 57% when used as first- and second-line treatment for group D and E disease. Shields et al. [**28**] conducted a study on six eyes with group D disease treated with IVC and IAC with a 67% success rate. Abramson et al. [**9**] reported successful results in 85.1% of the group D patients when treated with IAC as first-line treatment (n = 47). 

It may be possible to increase the globe retention rate with the earlier use of rescue IAC, as our early cases were treated as a last resort. Overall, the results of the present study and other studies showed that IAC is a powerful tool as an IVC adjuvant for group D and group E retinoblastoma, allowing the salvaging of more eyes compared to IVC alone, or with the use of focal therapy.

To assess whether certain factors of group D or group E eyes could determine a higher success rate of IVC or a higher risk for enucleation, we observed that none of the variables appeared as risk factors for enucleation. This is not surprising as, by definition, group D eye tumors have common characteristics such as tumor size, retinal detachment and seeding. In two cohort studies, group D and other ICRB groups were treated with primary IVC [**29**,**30**], so no analysis was performed for group D disease alone in this study. There have been no reports of specific risk factors for secondary enucleation of group D disease initially treated with IVC in literature.

Interestingly, in this study, eyes with single focal tumors and those with relatively larger horizontal corneal diameters (in both eyes) were at significant risk for primary treatment failure. We have no rational explanation for these observations. It is also worth noting that the diagnosis of numerous tumors, usually in cases of genetic disease and especially in group D disease, is not always possible as retinal detachment can limit a detailed ultrasound and exploration in most cases.

In 8 eyes with group D disease classified into treatment set I, there was no need for additional adjuvant treatment to achieve tumor control, whereas all other eyes received IVC in addition to adjuvants. Certain studies suggested that the success rate can be increased when the chemotherapy is used together with local treatment modalities [**31**], although the adverse effects of the focal therapy should be kept in mind [**32**,**33**]. 

The extraocular complications and side effects resulting from IVC and IAC in our study were comparable to those found in literature [**13**,**34**]. Among all cases, only two had an anaphylactic reaction to intravenous carboplatin, which was conservatively managed. 

Regarding disease metastasis, IVC has a protective role as compared to primary IAC [**8**]. Nevertheless, in a recent study, primary IAC achieved an 85% success rate and metastasis from the intraocular tumor was observed in 6% [**9**], implying that IAC success might lead to a higher risk of disease metastasis. Globe salvage rate in retinoblastoma group D was 75% and in group E 20.6%. Limitations of this study included its retrospective study design with analysis being done only for group D and E disease. Furthermore, 20.98% of eyes underwent upfront enucleation, therefore being excluded from the chemotherapy-based analysis. 

## Conclusion

Globe salvage rate for group D retinoblastoma was higher than in group E disease. Different treatment combinations of primary and secondary intravenous chemotherapy, as well as intra-arterial chemotherapy, demonstrated comparable success rates. Intravenous chemotherapy with adjuvant intra-arterial chemotherapy, laser therapy, cryotherapy and intravitreal Melphalan has shown safe and effective results and achieved an 83.33% globe salvage rate in group D disease and 60% in group E disease. Intra-arterial chemotherapy with adjuvant laser therapy, cryotherapy and intravitreal Melphalan may be a better option for globe salvage in group D and group E retinoblastoma.

**Conflict of interest**

The author(s) declare no potential conflicts of interest with respect to the research, authorship and/ or publication of this article.

**Informed Consent and Human and Animal Rights**

Informed consent has been obtained from all individuals included in this study.

**Authorization for the use of human subjects**

Ethical approval: The research related to human use complies with all the relevant national regulations, institutional policies, is in accordance with the tenets of the Helsinki Declaration, and has been approved by the Ethics Committee of Lahore General Hospital, Lahore, Pakistan.

**Acknowledgements**

None.

**Sources of Funding**

The author(s) received no financial support for the research, authorship and/ or publication of this article.

**Disclosures**

None.

**Equal contribution to the article**

Hussain Ahmad Khaqan and Rahul Rachwani Anil had an equal contribution to the article.
